# A Multi-facetted Visual Analytics Tool for Exploratory Analysis of Human Brain and Function Datasets

**DOI:** 10.3389/fninf.2016.00036

**Published:** 2016-08-23

**Authors:** Diego A. Angulo, Cyril Schneider, James H. Oliver, Nathalie Charpak, Jose T. Hernandez

**Affiliations:** ^1^IMAGINE, Systems and Computing Engineering, Universidad de los AndesBogota, Colombia; ^2^LCNS, CHULQuebec, QC, Canada; ^3^VRAC, Iowa State UniversityAmes, IA, USA; ^4^Kangaroo FoundationBogota, Colombia

**Keywords:** exploratory analysis, visual analytics, fMRI, MRI, tractography, cohort studies, python

## Abstract

Brain research typically requires large amounts of data from different sources, and often of different nature. The use of different software tools adapted to the nature of each data source can make research work cumbersome and time consuming. It follows that data is not often used to its fullest potential thus limiting exploratory analysis. This paper presents an ancillary software tool called BRAVIZ that integrates interactive visualization with real-time statistical analyses, facilitating access to multi-facetted neuroscience data and automating many cumbersome and error-prone tasks required to explore such data. Rather than relying on abstract numerical indicators, BRAVIZ emphasizes brain images as the main object of the analysis process of individuals or groups. BRAVIZ facilitates exploration of trends or relationships to gain an integrated view of the phenomena studied, thus motivating discovery of new hypotheses. A case study is presented that incorporates brain structure and function outcomes together with different types of clinical data.

## 1. Motivation

An important challenge in brain research, in both normal and pathological conditions, is to better understand the extent to which the physical structure of the brain influences its functioning. The most common research procedure is characterized by experiments aimed at collecting data directed toward testing a former hypothesis. This confirmatory-like methodology imposes limitations on the way data is used, and it is typically used only once which is unfortunate since data acquisition is generally time and resource intensive.

In the last two decades brain research data has increasingly been gathered in a more open fashion and many databases are now available to the public (Milham, 2012). In parallel, data collection, storage, and sharing, have been improved at both technical and policy levels, have advanced (Eckersley et al., 2003), together with technologies used to consolidate, search and access the data (Van Horn and Toga, 2009; Wood et al., 2014). This allows massive amounts of data to be consolidated into databases and searched in efficient ways. In this way questions can be explored and large data pools can be mined for interesting relationships.

The resting state functional magnetic resonance imaging (rfMRI) community is exemplary with large efforts such as the Consortium for Reliability and Reproducibility (CoRR) (Zuo and Xing, 2014) and the Human Connectome Project (Marcus et al., 2013; Hodge et al., 2016), which allow researchers to share data and enables the exploration of integrated datasets containing data from thousands of subjects.

This has led to rapid changes in the way research can be conducted, i.e., a shift from hypothesis-driven research into data-driven research, where data is available first and research questions and hypotheses are formulated on the basis of exploration of data. The methodology used in data-driven research differs significantly from that for hypothesis-driven research. Data-driven research seeks to find and extract meaningful insights from data using exploratory methods (Tukey, 1980) and has already proven effective in economics, terrorism prevention, and business intelligence domains which are characterized by large and heterogeneous data sets (Cook and Thomas, 2005). Exploratory research involves iterating through data several times, looking at it from different points of view, transforming data, searching for interesting subjects and measurements, gathering details and performing group analyses. These analysis tasks are carried out multiple times, and often in different order, as researchers learn more about the data. It is therefore helpful to provide tools to make annotations and save findings, so that explorations can be continued later as an integral part of the ongoing process of discovery.

During this process several data patterns may likely lead to unexpected insights. Unfortunately, it is also likely that these patterns are caused by the unique noise structure of the current data and therefore cannot be generalized to the global population. Automatic data-mining algorithms can find thousands of possible relations, but true findings need to be backed up by science and current knowledge. Therefore, domain experts must be involved in interpretation of insights. Moreover, insights that integrate data from different domains require experts from all these domains.

Visual analytics (Keim et al., 2008) has emerged as a discipline that seeks to integrate statistics, machine learning, data mining and interactive data visualization with the objective of optimizing the use of data available for exploratory research. The analyst is acknowledged as the most important actor, and all tools are designed to support exploration and provide timely access to the required data as well as to informatics and statistics functions. Another principle in visual analytics is that analysts should focus on data and not on operational details of the tools. Therefore, tools should provide the data and functionality to complete the task, while keeping non-relevant details and complex functionality hidden.

Exploratory brain research is a domain that could certainly benefit from visual analytics techniques. Indeed, brain function-related datasets are a combination of spatial (brain imaging) and non-spatial (clinical) measurements that could be analyzed together to better understand the link between brain structure and function as they relate to human health and behavior. Examples of spatial measurements include brain anatomy acquired by means of magnetic resonance imaging (MRI), neural pathways trajectory acquired by diffusion weighted imaging (DWI), patterns of cerebral activation in specific tasks and acquired by functional MRI (fMRI), or brain networks functionality and corticomotor function tested by transcranial magnetic stimulation (TMS) of motor and non-motor areas of brain. Specialized tools can process these neuroimaging modalities to model brain structure, build pathways, produce statistical maps of activation patterns, and neural connections. Brain researchers need to correlate these neurophysiological measurements and models to data of a different nature, such as neuropsychological performance, behaviors, and other clinical data.

However, the tools currently used in brain research are generally specific to the type or domain of data analyzed and they are optimized to support linear work flows. It follows that experts must often switch between tools to integrate and analyze data from different domains. In the worst cases, they may even have to move to a different computer. This process is time-consuming and repetitive. It requires the analyst to focus attention on the “how” rather than the “what,” and thus makes exploratory analysis challenging.

## 2. Introduction

This paper introduces BRAVIZ, a software tool based on visual analytics and aimed at supporting exploratory analysis in brain research. More specifically, it focuses on datasets that include MRI derived measurements and models as well as TMS and clinical outcomes. BRAVIZ is comprised of several applications that integrate interactive visualizations, links to detailed meta-data, creation of new variables as well as statistical models and analyses, all of which are designed to support and facilitate full exploratory analyses. It is implemented on python and available under an open source license.

## 3. Related work on neuroimaging and exploratory analysis

Several data processing and visualization tools are available to support research on neuroimaging. For example, Freesurfer (Fischl, 2012), FSL (Jenkinson et al., 2012), and SPM (Friston et al., 2006) segment, register and perform statistical testing of brain image data. 3D Slicer (Fedorov et al., 2012), Brain Visa (Cointepas et al., 2001), and ITKSnap (Yushkevich et al., 2006) are commonly used to integrate data from different image modalities (structural, diffusion-weighted, functional among others). They have all proven to be efficient at processing bulk images in a pipeline, and visualizing data from a single subject, but they fall short when several iterations through the data are required. The interfaces proposed for statistical testing require extensive configuration, which is appropriate for testing specific hypotheses, but becomes cumbersome when several possibilities are to be explored. Efficient mechanisms for restricting analysis to only a subset of subjects (e.g., with common clinical or lesion characteristics) or going back to a subject's details are missing. Complementary data loaded from tables can be used, but changing variables often means creating new tables and making them fit the required format.

Non-spatial information visualization tools like GGobi (Cook and Swayne, 2007) and Tableau (Hanrahan, 2003) can be used for interactive exploratory analysis. They support data transformations, model fitting, and interactive visualization. They also enable detection of outliers (important in data-driven research whereas usually unnecessary in hypothesis-driven research), provide additional details, determine subsets of subjects, and visualize patterns and trends in different ways (e.g., parallel coordinates, scatter plots, histograms, etc.). However, these tools do not integrate well with spatial data. Scalar data derived from original images can be added but there is no easy way to link back to the original data or to explore spatial features that cannot be encoded into numerical variables.

Recently, there has been an increased interest in resting state fMRI (rfMRI) and the connectivity networks that are inferred from it (Biswal et al., 2010; Rubinov and Sporns, 2010). Analyzing this data requires the mixed use of spatial tools performing voxel wise and cluster analyzes with graph oriented tools and statistical analysis tools. The Connectome Computation System (CCS) (Xu et al., 2015) integrates data pre-processing tools, with connectome generation and finally mining and visualization of automatic data. This kind of integrated environment allows researchers to efficiently explore data and pursue multiple hypotheses. BRAVIZ focuses on the last layer of this process but is focused on other neuro-image modalities.

INVIZIAN (Bowman et al., 2011, 2012a,b) uses Ggobi options to explore, in an abstract 3D space, the relations between scalar values and anatomical features of large brain datasets. It provides an environment for exploratory research involving data from several databases for hypotheses generation. However, it works only with automatic feature extraction from structural MRI. Like BRAVIZ, INVIZIAN focuses on easing the users' visual pattern search, however BRAVIZ targets a broader range of spatial data, and focuses on data generated by users rather than on machine learning.

A visual analysis tool for high dimensional genetic and clinical data is presented in Hinterberg et al. (2014). The user explores the data by quickly iterating through several models that relate genetic data to clinical outcomes. Models are displayed as trees and linked with distributions of the selected parameters. Tools are provided to automatically find the most relevant parameters to reduce the size of the search space. This tool is optimized for a single type of data, a single type of model, and a specific workflow. In contrast, BRAVIZ proposes to support multiple tasks, data types and workflows by providing a set of applications to be used independently or combined for complex exploratory analyses, as for example to interactively probe the multifaceted relationships between spatial and non spatial outcomes at the level of individuals, subsets of subjects or group of subjects.

Even though multiple tools exist for analysis and exploration of spatial and non-spatial data, the integration of multiple kinds/levels of analyses and data in a single environment remains challenging. BRAVIZ provides a unique unified environment for analyzing spatial and non-spatial data interactively, and in this way, it supports data-driven research.

## 4. BRAVIZ architecture

**Figure 1:**
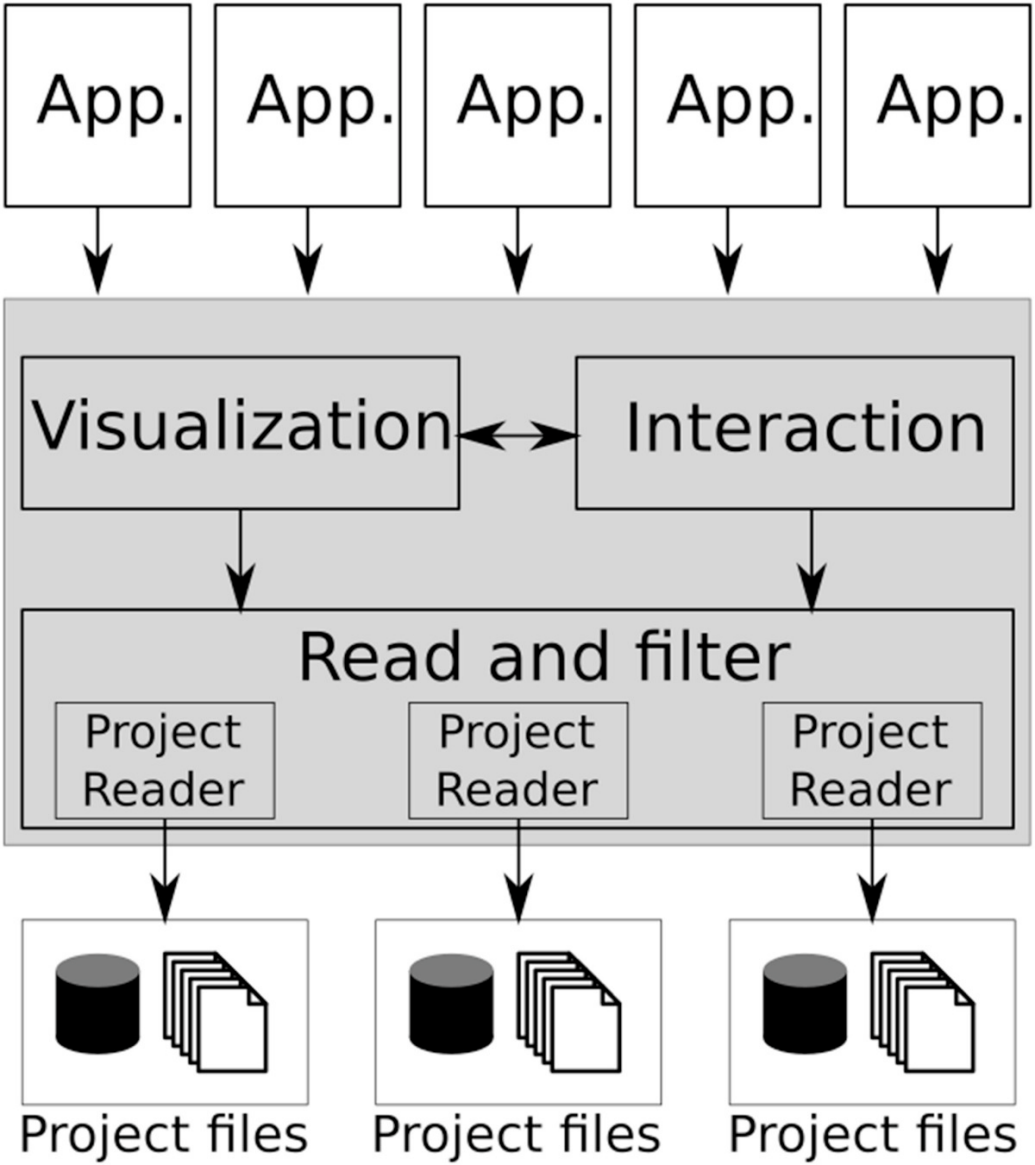
**The BRAVIZ software architecture, the main library is shaded**.

Figure 1 shows the BRAVIZ architecture which is based on the Model-View-Controller pattern, where the bottom layer (Project reader) together with the data repository constitute the model, the BRAVIZ Library (shaded region) makes up the controller, and each application represents a different view.

Instead of a large monolithic application, BRAVIZ takes a distributed approach and thus is comprised of a set of applications tailored toward specific analysis tasks and data types. New BRAVIZ applications are implemented based on the common library, freeing developers from thinking about technical details related to data manipulation. All applications use the same model, which also provides a channel for data sharing. Users can create custom samples, new variables and custom geometric structures and store them in the database, where they can be read by any other BRAVIZ application. An additional communication mechanism is provided that enables applications to exchange data in real-time. In this way individual applications can be combined to solve more complex tasks. Section 4.2 will describe current applications.

The library provides tools for loading spatial data (and transforming it into an appropriate coordinate system), for manipulating tabular data, for creating spatial and non-spatial interactive data visualizations, and for interacting with other applications and between different users. The types of spatial-data supported by the current implementation are: structural MRI, diffusion MRI, functional MRI, label maps, tractography reconstructions, structure segmentation models, and Freesurfer cortex reconstructions. Non-spatial data can be any numeric or categorical variable, including TMS, clinical, and socio-economic data. More details on the Braviz library will be given in Section 4.4.

### 4.1. Design and development methodology

A “user centered” approach (Wassink et al., 2009) was taken to design and implement BRAVIZ. The authors worked closely with brain researchers of several specialties, visited several labs and hospitals, and learned as much as possible about research workflows and the bottlenecks they contain. Prototypes were implemented in several iterations and shared with different domain experts, whose feedback motivated the design of the next generation. The initial stages of design focused on identifying the obstacles that affect exploratory analysis and communication between experts, and examined ways of mitigating them. The team of experts was composed of radiologists, psychiatrists, physicians, neurophysiologists, physical therapists, pediatricians, statisticians, engineers, and economists.

From analyzing the visualization options in current neuroimaging tools, as well as how domain experts used them, the BRAVIZ team learned what was expected from image viewers, i.e., which features were important to implement and which were seldom used. SPM (Friston et al., 2006), Osirix (Rosset et al., 2004), and 3D-Slicer (Fedorov et al., 2012) were the reference at this stage. For example, researchers needed to visualize several types of data in the same space and to be able to compare brain images between two subjects. Also obvious was the need to integrate spatial visualizations with non-spatial data for the same subject in order to be able to understand relationships. Another common issue discovered in this exchange with expert users was that navigation from one subject to another typically requires re-starting the visualization application which is cumbersome.

In addition, there was the need for performing statistical analysis in real time, working with different groups of subjects in the same space, creating and importing new data on the fly, performing group analyzes, identifying outliers, and detecting data quality issues.

### 4.2. Applications set

**Figure 2:**
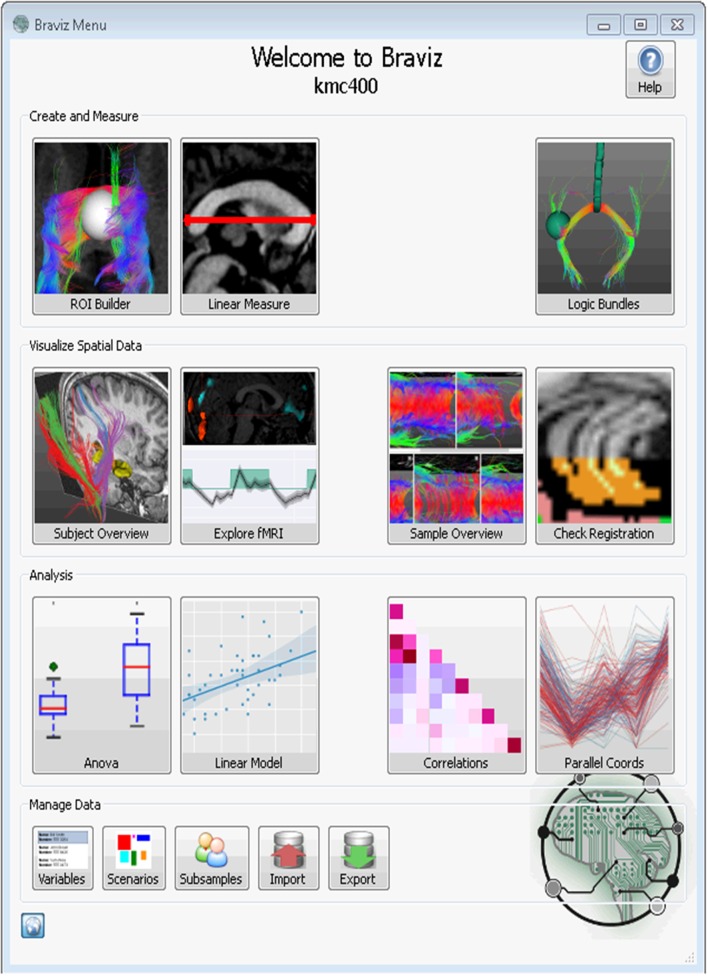
**BRAVIZ main menu, each button provides access to an application**.

The current set of BRAVIZ applications can be divided in three categories. One set of applications measures or creates new descriptors from geometric data, one displays geometric data using other variables as context, and the third explores numerical and categorical data. This supports different stages of the exploratory analysis process, respectively, data transformation, visualization, and subjects-to-group analyses. These applications are accessible from the main menu (Figure 2) by clicking on the corresponding buttons. The bottom row provides access to utilities to manage samples, variables and scenarios, as well as importing and exporting data.

#### 4.2.1. Descriptors for geometric data

**Figure 3:**
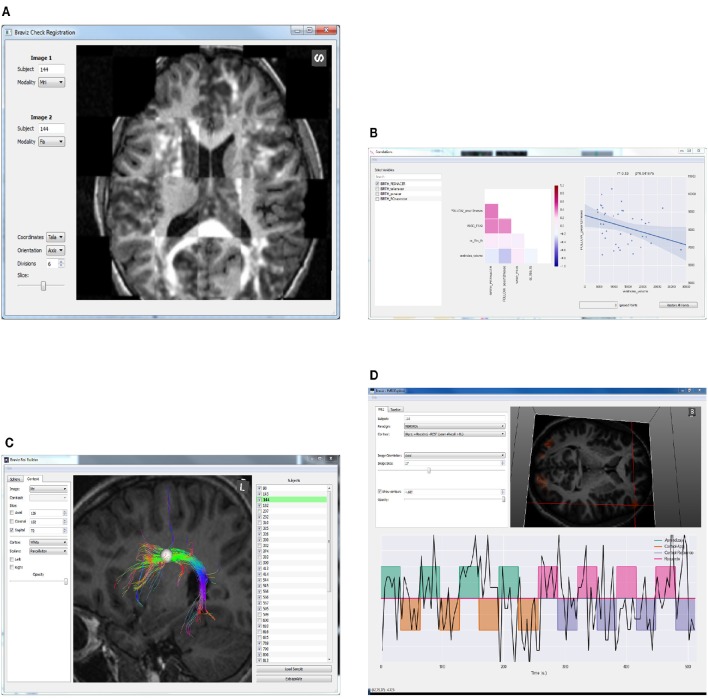
**Examples of BRAVIZ applications. (A)** Check Registration, **(B)** Correlations, **(C)** ROI Builder, **(D)** FMRI Explorer.

The *Region of Interest (ROI) Builder* application (Figure 3C) provides an interface for helping experts position a spherical ROI within the brain of each subject. These spheres are placed and sized with respect to images (from any modality) or cortex reconstructions. It is also possible to preview the fibers that cross the ROI, and to evaluate mean value inside the sphere of any scalar image, for example mean FA (fractional anisotropy associated to the integrity of myelin covering axons) or mean *t*-value associated with an fMRI test (representing to what degree a region is involved in a given task). Linear and non-linear registration maps can be used to approximate the position and size of the sphere in other subjects. The expected workflow is positioning the ROI in one subject, extrapolating it to a sample and making fine adjustments in position and size. The interface is optimized to support this (buttons, hotkeys, and visualization). Several ROIs can be used to select specific or more complex bundles. All data generated in the application (ROIs, bundles, and scalars) can be used on any other BRAVIZ application.

In addition, the current BRAVIZ implementation includes an application for making linear measurements (lengths) of brain structures, and an application (*Logic Bundles*) for defining fiber bundles by combining ROIs and segmented structures through logical operations. For example, a bundle may be defines as the fibers that go through structure *A* or ROI *B* but that don't cross structure *C*.

#### 4.2.2. Geometric data visualization

**Figure 4:**
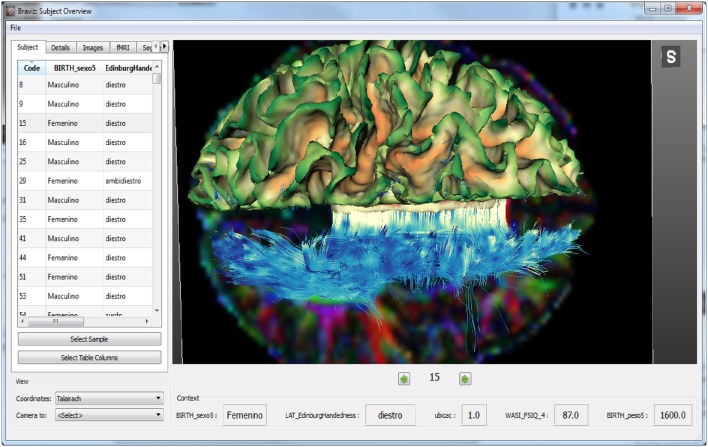
**Main interface of the subject overview application: at the bottom of the render some variables about the subject provide context**.

The *Subject Overview* application is shown in Figure 4. This tool provides access to several kinds of spatial data in a unique 3D renderer and eases the rapid navigation of the data from one subject to another. Geometrical features such as MRI volumes or mean fractional anisotropy of DWI in relation to a chosen structure can be captured directly from this application and added to the database as a new variable. The application also shows the values per subject of selected variables as well as users' annotations. This ensures an integrated overview of each case and follow-up between different users depending on the data available to BRAVIZ.

**Figure 5:**
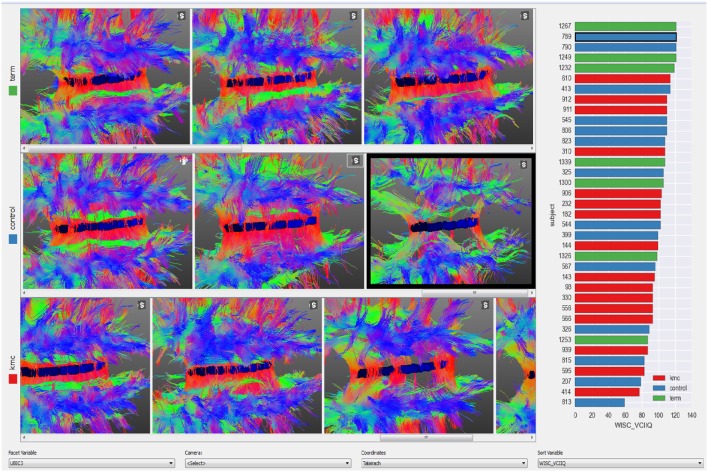
**Quality control on the corpus callosum fibers from the whole sample**.

A small multiples display (Tufte and Graves-Morris, 1983) with views of several subjects is useful for finding trends across a sample or for quality control to detect contaminated images. Subjects are ordered from left to right according to a chosen numerical variable and each row corresponds to a nominal variable. In the example presented in Figure 5 the three rows are children born at term, preterm in incubators (preterm controls) and preterms with Kangaroo Mother Care intervention (see case studies in Section 5) and subjects are sorted from left to right according to the score on an IQ test. The bar-plot at the right shows the distribution of this test amongst the three groups. This plot is linked with the 3D views, thus clicking on a bar will bring the corresponding subject into view. If more details of any subject are required, the user may right click on that subjects image to load the corresponding subject on other BRAVIZ applications.

The *fMRI Explorer* application from Figure 3D displays raw BOLD signals and contrast designs associated with fMRI experiments. It can also be used to compare signals at different locations or from different subjects. The *Check Registration* application (Figure 3A) allows the user to visualize simultaneously two images, from different modalities or different subjects and in a given coordinates system to assess the quality of registration.

#### 4.2.3. Numerical and categorical data exploration

**Figure 6:**
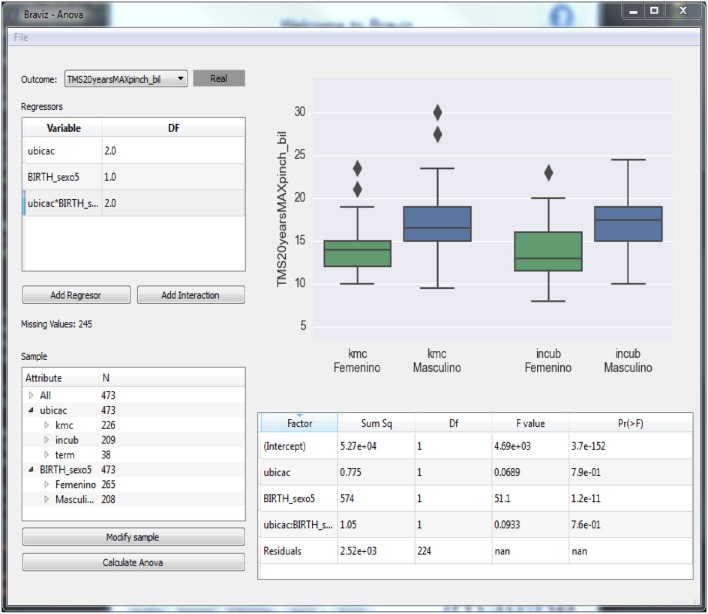
**An application for performing ANOVA analyses using the data in the BRAVIZ database**.

The *ANOVA* application (see Figure 6) provides access to statistical models implemented in *R* (Team, 2012). In order to fit a model the user has to use the left side panel to select the outcome variable, the regressors and interaction terms and the sample. Variables are selected from the database, which additionally stores the type of each variable, its description and in the case of nominal variables the labels for each level. The variable selection dialog shows this meta-data for each variable and allows users to modify it. It also displays an overview plot of the variable which allows the user to infer the distribution of values. This plot can be configured to show the relationship between two variables (outcome vs. regressor) so that the user may do a preliminary visual assessment of relations between variables. Finally, the dialog provides mechanisms to search the database. When all parameters are set the user clicks the *Calculate ANOVA* button, the system will fetch the variable values for the selected sample from the database and fit the model using the *CAR* package of R.

After fitting the model, the main plot will show diagnostics (distribution of residuals and a scatter plot of residuals vs. fitted values) for the validation of the ANOVA hypotheses (noise normally distributed and with constant variance) and the table at the bottom right shows the resulting statistics. The application can also show box-plots and scatter-plots that provide additional insights on the relation between regressors (nominal and numeric variables and interaction terms) and outcomes. Individual points in these plots can be identified by positioning the mouse over them. Additionally, by right clicking the mouse, the associated individual subject data can be loaded into other BRAVIZ applications to get supplemental information. This can be especially useful for instance to interpret outliers.

**Figure 7:**
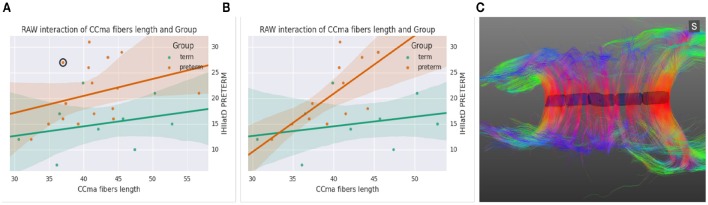
**(A)** Scatter plot and linear fit between the Ccma fibers and interhemispheric transfer timer, **(B)** same plot after removing four outliers, **(C)** corpus callosum of the circled outlier of plot **(A)**.

Figure 7 shows an alternative analysis application (*Linear Model*) which fits standard linear models and shows the effect of each regressor (numerical variables, dummy variables representing levels of nominal variables, and interaction terms). The application provides a similar interface and functions in the same way as the ANOVA application. The example from the figure shows a linear regression between the average length of fibers going through the mid-anterior section of the corpus callosum and the latency in Transcraneal Magnetic Stimulation test (see case studies in Section 5).

The *Correlations* application shown in Figure 3B displays a list of variables, a correlation matrix of selected variables, and scatter plots of selected correlations. Points in the plot can be queried and right clicked. Additionally, they can be temporarily eliminated from the analysis to see the impact they have on the correlation that is currently tested.

**Figure 8:**
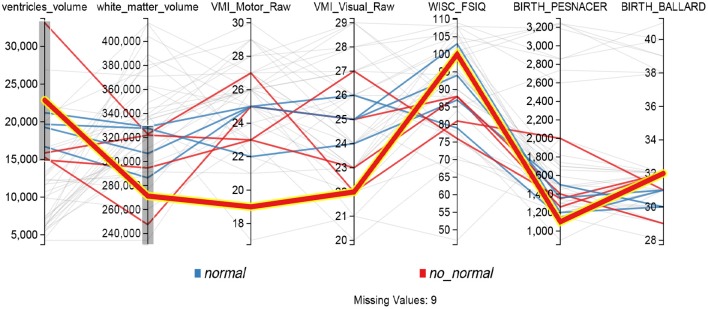
**Parallel coordinates display configured for searching possible PVL cases, lines that don't match the filters on each axis are shown in gray**.

The *Parallel Coordinates* display from Figure 8 provides another way of analyzing relations involving several variables. This functionality is exemplified in the second case study presented thereafter (see Section 5). Each vertical axis represents a variable, and each line is a subject. Users can interactively apply filters to each axis, in order to see how changes in one variable affect other values, and to understand relations involving several variables. In the figure, gray lines represent subjects excluded by the filters while color lines are those subjects that match the filters.

### 4.3. Global features

In addition to the unique features of each BRAVIZ application, the integrated platform provides features and combinations of tools (designed to be used together) in order to specifically favor the complex process of exploratory analyzes.

#### 4.3.1. Linking clinical outcomes and brain data

Subjects in BRAVIZ are an integral part of the process, and therefore all types of data per subject is always accessible. For example, the *Subject Overview* application displays a set of clinical variables and annotations together with spatial data. It follows that users of BRAVIZ can make a context-dependent reading of images and of other spatial data. Such information would only be displayed elsewhere by other tools.

Several scalar measurements can be derived from spatial data within the tool itself. For example a group of segmented structures can be selected and their combined volume added as a new variable. These values can afterwards be used together with clinical variables for statistical analysis, but a link is kept between the initial data and the environment in which it was generated. Continuing with this example, if the researcher finds an outlier, she/he can right click on it and from the context menu open the application in which the odd value was generated. The application will load with the configuration it had at that time, but focused on the subject of interest. In this way the extreme value can be analyzed to determine if it is caused by a particular artifact of the subject, such as a segmentation error, a problem with the image, or a sign of an actual pathology.

#### 4.3.2. Comparing subjects

A common task in exploratory research is analyzing similarities and differences amongst a group of subjects. In most existing visualization tools for spatial data, users have to select the subject of interest at the onset of analysis, and then configure all the visualization options and load the necessary files. BRAVIZ takes a different approach, and always allows the subjects of interest to change in the middle of the analysis while maintaining the configuration of the application. In this way it is easy to look at different subjects from the same point of view, which makes comparing data very efficient.

#### 4.3.3. Working with samples

Often some properties of the data apply only to a specific group/sample of subjects. In BRAVIZ samples are a central component of every analysis. Samples can indeed be defined by using filters on variable values, manually adding and removing specific subjects, taking random subsets, or combining samples through set operations (union, intersection, and difference). These samples can then be used for iterative analyses between different groups or can be modified (e.g., withdrawal of outiers), and the results of the modification visualized in real time. This contrasts with hypothesis-driven research where the sample must be set at the onset of analyses.

#### 4.3.4. Working with incomplete data

It is not uncommon for some values or geometric data to be missing in some subjects. Applications that show geometric data detect missing data, hide the corresponding object from the scene, and display warning in the status bar. Group analysis tools display the number of missing points, and ignore them when performing calculations. Also, when building samples, the using filters the interface provides a checkbox that allows the user to include or exclude subjects with a missing value for the specific variable studied.

#### 4.3.5. Supporting long workflows

Analyzing a complex dataset requires a significant amount of time, and will likely be split in multiple sessions. BRAVIZ applications allow saving analyzes and restoring them using custom names and descriptions, and attaching textual annotations to subjects, variables, and geometric objects (for example ROIs), and thus favors re-use of previous explorations. A log of each analysis session is kept, which can be reviewed and annotated using a web interface. These features allow other users to understand the meaning of variables, geometric structures and scenarios created by colleagues thereby favoring collaboration.

#### 4.3.6. Integrating tools

**Figure 9:**
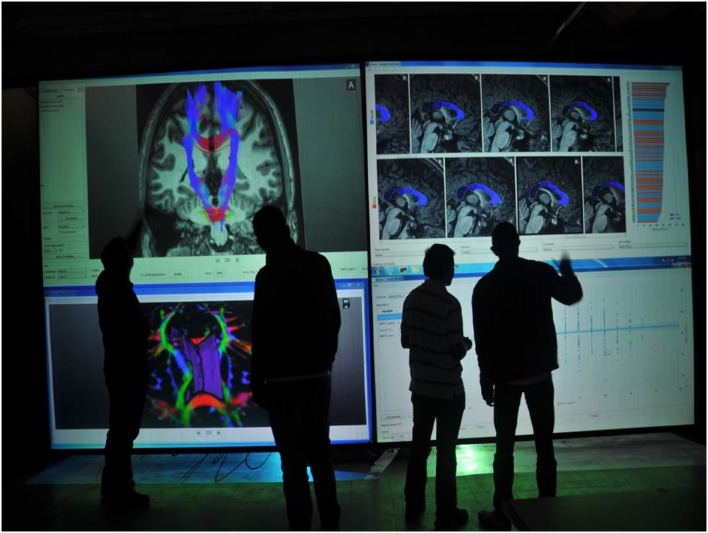
**An example of BRAVIZ running on a large display in a collaborative setting**.

In addition to the common database where all tools read and store variables and geometric objects, BRAVIZ includes a real-time communication mechanism. All applications can be coordinated to focus on the same subject (see Figure 9), to work with the same sample, or use the same set of variables. Applications may be running on different screens or even different devices; allowing users to get different perspectives of the data at the same time. Of note, BRAVIZ applications have the option to block specific parameters to keep the application from changing if the user prefers it. Also, for some actions that are likely to produce important changes, BRAVIZ asks the user for confirmation, and provide the option to always accept or always ignore the specific change.

Multiple users can work with the same data base thus can share data and thoughts. In this way one user may create a new measurement and make it available to other users. Real-time communication is implemented through TCP, therefore it would be possible to link together multiple workstations, but the research team has yet to explore whether this would provide a convenient user experience.

### 4.4. The BRAVIZ library

The Braviz library (shaded region of Figure 1) is divided in three modules that provide several common features to ease the development of applications. In addition, it abstracts access to data so that applications can be ported to different datasets. Of note, BRAVIZ library can also be used in python applications and scripts, including interactive work in a terminal or an iPython notebook.

#### 4.4.1. Read and filter

The *Read and Filter* module is in charge of reading the configuration file and instantiating the appropriate project reader class. In addition, it holds several utility functions for converting image data between formats, applying affine transformations, filtering tractography, and deriving scalar measures from scalar data. This module also abstracts reading and writing data from the BRAVIZ database. Currently this database is implemented in *SQLite* (Hipp et al., 2015) and it holds all non-spatial data, samples, scenarios, and other objects created by users.

#### 4.4.2. Visualization

The Visualization module contains functions and widgets that can be used to create spatial and non-spatial visualizations. Spatial visualizations are implemented with VTK (Schroeder et al., 1996), but several classes are available to streamline development. For example, *managers* are available for tractography, fMRI, and segmentation data. These are high-level classes that connect to the *Read and Filter* module, load the appropriate data, and manage the VTK visualization pipeline ending in a specified renderer. The user only needs to provide the current subject, current coordinate system, and visualization parameters. Of note, these classes expose methods as *change_subject* and *change_coordinates* which conveniently allows switching to a different subject or a different coordinate system.

Non-spatial visualizations use Matplotlib (Hunter, 2007) and Seaborn (Waskom et al., 2014), or D3 (Bostock et al., 2011) in case of web based applications. This module provides functions to create common visualizations by providing an input data-frame and other configuration parameters. All BRAVIZ visualizations exhibit consistent behavior, i.e., they allow the user to query the subject id of a data point by hovering over it, create a context menu by right clicking on a point, and allow for highlighting points on the display.

#### 4.4.3. Interaction

The *interaction* module includes common QT widgets as well as utilities to create web based applications and to handle communications between applications. It also handles connection to external tools. For example, it contains functions that use the *R* system to perform statistical analysis.

### 4.5. Importing data into BRAVIZ

As mentioned above, making BRAVIZ generalizable to support different data-sets and data-formats was a primary design goal. In order to use BRAVIZ on an existing data-set, spatial data should be pre-processed using standard neuroimage tools. Instead of copying spatial data, BRAVIZ access it using specific *ProjectReader* classes. Finally several options are available to import non-spatial data.

#### 4.5.1. Pre-processing

While BRAVIZ integrates some basic image processing algorithms, the focus remains on interactive visualization. In this end, data should be pre-processed using third party tools before starting a BRAVIZ project. As a minimum, BRAVIZ expects registration matrices linking the different coordinate systems present in images. Running the FreeSurfer *recon_all* pipeline will provide BRAVIZ segmentation, cortical parcellation and Talairach registration, which are basic for most visualizations.

In addition, BRAVIZ can read SPM statistical maps and warp fields as well as tractography output (currently in VTK format), Tracula bundles and any type of scalar map, for example those derived from the tensor model.

Pipeline systems such as LONI pipeline (Dinov et al., 2009), NiPype (Gorgolewski et al., 2011), or the CCS (Xu et al., 2015) are ideally suited to automate these necessary pre-processing steps.

#### 4.5.2. Reading spatial data

BRAVIZ accesses geometric data through the project reader interface (see Figure 1), which can have different implementations for different projects. All the upper layers, including applications, will access data through *ProjectReader* objects by specifying the type of data, the subject id, the coordinate space, and additional parameters appropriate for each data type (e.g., scalars for tractography or contrast for fMRI maps). This object is also responsible of returning indices of available data. Internally the class must be able to load the appropriate data, apply the correct transformations, and finally return the requested data as a python object.

For example, in the case study presented below, all project's data was stored on hard disk, using NIFTI format for images, and VTK format for tractography and segmented structures. Files were stored in a layout such that the full path for each file can be determined based on subject id. Pre-calculated transformation matrices and warp fields were located and internally applied on load.

New reader classes can be implemented to read data from different sources and different formats (e.g., reading DICOM objects from a PACS). The nibabel and vtk python libraries provide functions to load data stored in several formats, making the implementation of these classes feasible in a short amount of time.

It is noteworthy that setting up BRAVIZ to work with a new dataset requires expertise from engineers. Recall that BRAVIZ is targeted at interdisciplinary teams and one of the goals is allowing the members of these teams to work together efficiently. Additional details of how BRAVIZ handles spatial and non-spatial data are described in the project's documentation.

#### 4.5.3. Importing non-spatial data

By means of integrated tools clinical data can be imported into the system from excel tables or SPSS files using integrated tools. This data is copied into a database, and can be accessed from all applications. The python terminal or iPython notebooks (Prez and Granger, 2007) can also be used to interactively read data from other sources (for example scraping web sites or by parsing DICOM headers), transform it into a pandas dataframe, and save it into the BRAVIZ database using a high level API.

Of note, additional data can be added at any time and non-spatial data can also be exported to an spreadsheet using a graphical interfaces. It follows that the user is able to take some variables of interest out of BRAVIZ, perform calculation on an external tool, and import the results back into BRAVIZ.

## 5. Case studies

The functionality provided by BRAVIZ can be used both for untargeted, intuitive and adaptive discovery of relationships in order to improve understanding of data; as well as for structured dissection of data. In Section 5.1 an example of adaptive data exploration is presented. Section 5.2 illustrates pre-structured, targeted exploratory analysis to identify potential candidates for a sub-analysis of leukomalacia in a cohort of adolescents followed since birth.

In these case studies, MRI, fMRI and diffusion data were acquired, anonymized, and stored in DICOM format, then pre-processed using dcm2nii (Rorden and Brett, 2000), fsl (Jenkinson et al., 2012), freesurfer (Fischl, 2012), camino (Cook et al., 2006), and spm (Friston et al., 2006). A custom BRAVIZ project reader was implemented to read the output of this process. Clinical data was primarily consolidated in an SPSS database then imported in BRAVIZ. These tasks were performed by the engineering members of the group. Afterwards, researchers were free to explore and analyze data with BRAVIZ on their own computers. The first case study was carried out by a neurophysiologist, while the second one was completed by a pediatrician and a neuro-radiologist.

### 5.1. Adaptive exploration of anatomical and functional data in prematurity

The collection of the data used in the case studies was approved by the ethical committee from the school of medicine of Universidad Javeriana (Bogotá, Colombia) as well as the ethical committee of Fundación Santafé de Bogotá (Bogotá, Colombia), all participants and their parents provided written informed consent. This section describes an adaptive inference process in which insights inspire subsequent exploratory steps, and, more specifically, highlights the flexibility that BRAVIZ affords.

#### 5.1.1. Rationale and initial hypotheses

Data comes from the seminal demonstration (Schneider et al., 2012) of the influence of a very premature birth (<33 weeks of gestational age) on brain function in a sample of 15-year old adolescents who were compared to their term peers. This study also showed the positive impact of an early care protocol (Kangaroo Mother Care) on brain functions, but this is beyond the present topic. The authors used the noninvasive and painless TMS of the primary motor cortex (referred to as the motor brain) and showed that the control of hand by brain was suboptimal in the preterm group. Especially, the excitability of the motor brain was lower and the time required for the transfer of nervous signal from one cerebral hemisphere to the other (interhemispheric transfer) was longer. These abnormalities were related to the deleterious after-effects of a premature birth that could still be detected 15 years later in brain (Schneider et al., 2012). Indeed, the interruption of the *in utero* maturation of the corpus callosum, a structure comprised of large-diametered fast-conducting myelinated fibers and connecting the two cerebral hemispheres, interfered with the normal installation of circuits in each hemisphere and of the functional lateralization between hemispheres, thus altering the sensorimotor and cognitive brain function (Schneider et al., 2008; Flamand et al., 2012; Schneider et al., 2012).

However, the study had not yet established any link between MRI-DTI measurements of the corpus callosum thinning and the lower clinical performances in the preterm group as compared to the peers term. Two reasons can explain this limitation: the difficulty to statistically analyze, visualize, explore, and understand the link between outcomes of different nature (severely time-consuming with the use of different softwares and data transformations likely providing biases or mistakes); the required involvement of experts dedicated to the interpretation of each outcome.

The working hypothesis was that BRAVIZs user-centered approach and applications/modules with real-time statistical analyses (color coding for instantaneous detection of a correlation for example), and access to multifacetted neuroscience and clinical data (spatial and non-spatial interactive data visualizations with common operations available between applications) should favor multi-tasking along with adaptation to user's expertise leading to easy detection of outliers and emergence of unsuspected relationships between target variables.

#### 5.1.2. Contribution of BRAVIZ to scientific discovery (ancillary hypotheses)

All data had been already imported in BRAVIZ by the engineer before onset of work and this substantially fastened the access to spatial and non-spatial data. BRAVIZ was used to explore the potential links between clinical outcomes and lower excitability of the motor brain, interhemispheric dysfunction, and volume/numbers of fibers of the corpus callosum. The applications were used following the non-linear procedure that BRAVIZ uniquely supports. ANOVA results with scatter plots, standard deviations and superimposition of individual values helped immediately detect four outliers in the 15-year preterm adolescents sample. This should have taken far more time with other applications not processed by BRAVIZ in real time and in parallel. The *Correlation Viewer* allowed to remove these outliers with a simple click and to see instantaneously that they affected ANOVA results, which would not have been possible to detect if all applications had not be launched in the same workspace. Thus, the combination of *ANOVA, Correlation Viewer*, and *Parallel Coordinates* (MRI for structure volume, DTI for fibers) showed that, without the outliers (studied below), the medial-anterior part of the corpus callosum (maCC connecting the motor areas between hemispheres) was smaller in volume in the preterm group (17, 228 ± 5100 mm^3^) than in the term (22, 352 ± 3900 mm^3^; *p* = 0.02) and exhibited fewer callosal fibers (1806 ± 573) than in the term (2600 ± 570; *p* = 0.008). Despite this thinning of corpus callosum, no link was detected with the interhemispheric time of transfer between motor areas that was shown to be significantly longer in the preterm group (Schneider et al., 2012). However, the correlation matrix and scatter plots brought to attention that, by excluding the four outliers, a strongly red-colored (significant) link appeared between the increasing interhemispheric transfer time and the longer length of the maCC fibers: Figure 7B shows this strong correlation for the preterm group (orange circles) when the outliers are removed (as a contrast to Figure 7A), the longer the maCC fibers and the longer the interhemispheric transfer time. This correlation was absent in the term group (green circles).

The real time access to clinical characteristics by right clicking the dots in BRAVIZs scatter plots eased to understand that outliers in Figure 7B were participants with either cerebral palsy or Xfragile pathology. The parallel access DTI data and scatter plots in the same workspace favored the immediate detection that the circled outlier in Figure 7A was a participant with a dramatic thinning of the maCC (Figure 7C). This explains that, as compared to other participants with shorter length of maCC fibers, the interhemispheric transfer time was longer (holes in corpus callosum likely leading to temporal dispersion of current between hemispheres). We already knew that the preterm group had longer interhemispheric transfer time (Schneider et al., 2012) but BRAVIZ guided the analysis to rapidly visualize and test that this was related to the length of fibers in the preterm group, even if length was not different than in the term group. This contributes to raise the very new hypothesis that the underlying problem in our preterm sample may not not be the structural defect (excluding the outliers) but more likely the synaptic arrangement of connections between hemispheres, those with longer interhemispheric transfer time presenting with less efficient transcallosal networks.

Precisely, not all participants in the preterm group presented with lengthened interhemipsheric transfer time but five of them were over 20 ms when 8 and 14 ms are usually expected for men and women, respectively (Schneider et al., 2012). BRAVIZs parallel coordinates of the different variables was useful once again to efficiently identify that four out of these five participants over 20 ms had a transient score at the infant neurological international battery (Infanib) at 6 months of age. This intuitively led to launch BRAVIZs *Correlation Viewer* and to highlight a strong link between Infanib at 6 months of age and our neurophysiological measures known as different in the preterm group (Schneider et al., 2012) such as the lower excitability of the motor brain and higher occurrence of ipsilateral muscle responses to TMS of the motor brain (which is abnormal given the typical crossed organization of motor systems for hand function). In that vein, BRAVIZs applications contributed to highlight that the four participants above 20 ms thus who significantly drove the correlation in Figure 7A had a dysfunctional organization of the motor brain. One another ancillary hypothesis could be that Infanib testing at 6 months of age may be predictive of long-term impairment of the motor brain function, including a poor efficiency of the interhemispheric connections (time over 20 ms at 15 years of age, see Figure 7A).

**Figure 10:**
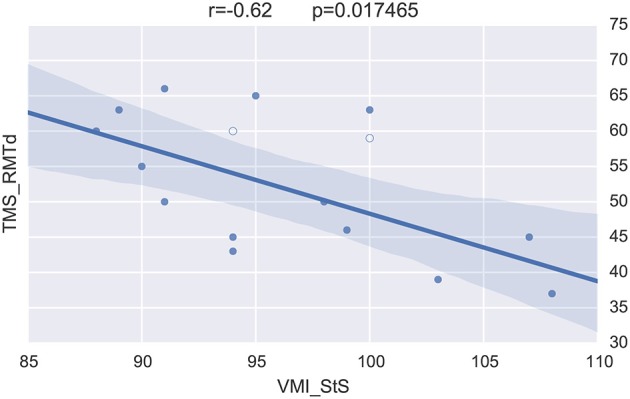
**VMI vs. M1 excitability excluding outliers (empty circles)**.

**Figure 11:**
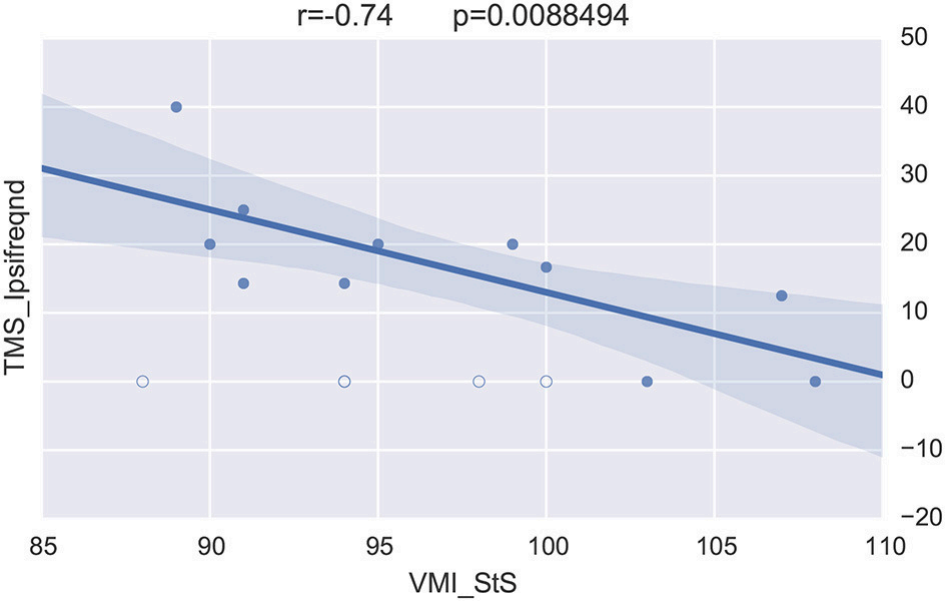
**VMI vs. ipsilateral corticospinal responses excluding outliers (empty circles)**.

Merging the neurophysiological expertise with the potential of BRAVIZ for data-driven analysis led to one last interesting new finding in this case study. This concerns the visuomotor control of movement which involved the corpus callosum function (Schneider et al., 2008) and which is assessed by the standardized score of the visuomotor integration test (VMI StS). This score was found to be significantly lower in the preterm group (95.5 ± 7) than in the term (108.5 ± 3.5; *p* = 0.0001) at 15 years of age. The *Correlation Viewer* (color codes highlighting the significant links and excluding the outliers determined above) used together with the parallel coordinates application (see all studied variables in real time in the same workspace) detected that the lower (lesser) VMI StS scores of the preterm group could be explained by a lower excitability of the motor brain (higher motor threshold, i.e., higher intensity of TMS to generate a response in the muscle, RMTd in Figure 10, the excluded outliers are represented by empty circles) and higher occurence of abnormal ipsilateral responses (Ipsifreqnd in Figure 11). By extension, BRAVIZ afforded applications and context to detect that the impairement of visuomotor control of movement in our sample of 15-year adolescents born prematurely was related to dysfunction of the motor brain and pathways, in terms of maladaptive synaptic organization (rather than anatomical issues), and that could have been predicted by the transient Infanib at 6 months of age. Overall, these new hypotheses driven by BRAVIZ in our sample of preterm adolescent may impact early rehabilitation when synaptic issues are more sensitive than anatomical to training-induced mechanisms of adaptation and motor learning.

#### 5.1.3. Conclusions

BRAVIZ adapted and intuitively guided the exploratory analysis of these multi-kind and multifacetted outcomes. With different experts gathered (i.e., neonatologist, neurophysiologist, psychologist, physical therapist) this new tool helped unmask important relationships between variables of brain function and uncover new hypotheses in prematurity that go beyond previous works in preterm children (Schneider et al., 2008, 2012; Flamand et al., 2012). BRAVIZ-related analysis of experimental data in prematurity pointed out for the first time that the long-term impairments (clinical data) in prematurity may not be the smaller volume of some structures but the lesser efficacy of synapses between neurons and of networks between structures (such as the corpus callosum between hemispheres). On that vein, a specific focus, in terms of quality and intensity of intervention for synaptic efficiency, should be made on those individuals presenting characteristics that BRAVIZ unique exploratory analysis has identified at risk for the integrity of brain function.

### 5.2. Pre-structured, targeted search, and characterization of leukomalacia patients

Periventricular Leukomalacia (PVL) is characterized by a lesion in the periventricular germinal matrix which results in a loss of white matter ventricular dilation. Preterm babies, especially those born prior to 33 weeks of gestation (due to non-invagination yet of highly vascularized germinal matrix) present with a significant risk of suffering from this condition and from its long-term physiological consequences, including motor and vision impairments, cerebral palsy and epilepsy.

Of note, the imaging protocol of the study did not include FLAIR images which are most appropriate for PVL detection. BRAVIZ and its applications thus offered the alternative to identify in the database for subjects who might possibly suffer this PVL condition. Such exploration targeted a better understanding of the samples health issues rather than directly testing a scientific hypothesis. However, incidental results may result from BRAVIZs data-driven analysis.

A parallel coordinates display was used to visualize the ventricles volume and the total white matter (myelinated fibers comprising brain networks). Figure 8 shows for example that the dataset included motor and visual testing, intelligence assessment, a classical neurological evaluation to discriminate between normal and abnormal neurological status and some risk factors, such as weight and gestational age (Ballard) at birth. Data could be filtered by axis dragging in order to keep only subjects with high ventricular volume and low white matter volume (shown in color at the figure, while excluded are shown in gray), i.e., with characteristics related to PVL condition. Lines (one line per participant) with decreasing slope from first segment (ventricules volume) to second segment (white matter) may reflect ventricular dilation (first segment) as a result of the loss of white matter, in relation to neurological status (abnormal in red and normal in blue).

**Figure 12:**
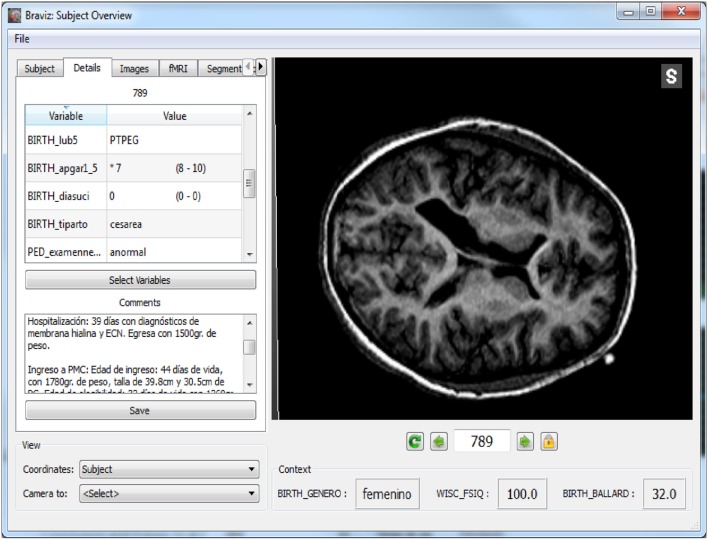
**Details of a subject with possible PVL, the left panel shows values for several variables as well as the full clinical history**.

The line highlighted in Figure 8 showed ventricules dilatation, low white matter volume, low scores for motor and visual components of visuomotor integration testing (VMI) and low birthweight (pesnacer). The context menu that appeared by right clicking on the line enabled to switch to the individual's brain facts, images and statistics in other BRAVIZ applications and to get additional details for better understanding the whole clinical profile of such an abnormal condition at 15 years of age. For example, the Subject Overview application denoted that the participant was born by C-Section in emergency after a twin pregnancy; the mother had HELPP syndrome (malignant hypertension) and died after giving birth; the abnormal status during the first year of life transformed in a diagnosis of cerebral palsy at the end of the first year (spastic diplegia and right arm hemiparesis). Altogether, the clinical profile and the symptoms together with the T1 image of ventricles dilatation (Figure 12) could be associated with PVL.

The 15-year subject data associated with the top line in Figure 8 (highest ventricles volume) showed in Subject Overview a very premature birth at 31 weeks of gestational age and given by C-section in emergency because of profuse bleeding (placenta previae), signs of leucoencephalopathy and an increased volume of the left lateral ventricle in CT scan, epilepsy not yet controlled, severe bilateral hearing loss, and abnormal bilateral vision, but a normal psychomotor development. BRAVIZs coordinated applications looking at indicator variables then at candidates' detailed profiles detected two cases with possible existence of PVL out of our sample of participants, although the original data had not been collected with the view of evaluating such white matter disorders. This witnessed the usefulness of the *Subject Overview* application to provide sufficient information to better understand each case and its life trajectory (variables, annotations) with no need to gather additional files.

## 6. Discussion

The cases studies illustrate how BRAVIZ can be used to improve understanding of a real data-set. A direct access to data makes researchers more efficient in the exploratory analysis and the recovery of data relevant to their questions. By combining several tools, users can perform group level analyses without loosing track of individual subjects, while getting additional details on a participant via a simple click. Sharing samples, variables, visualizations, and subjects of interest also provides an efficient channel for experts to communicate and share ideas, thoughts, advice, and opinions with each other.

Direct access to data helps save time and more interestingly, enables researchers to efficiently pursue and address in real time all questions coming to mind. If testing an idea, hypothesis of relationships or finding an answer requires a cumbersome procedure and gathering data from several sources, it will likely not be explored. This exploration would take non intuitive manipulation of different softwares and far more time with elevated risks of biases and mistakes in data transformation, etc. With BRAVIZ, the number of questions that can be addressed are increasing and so are the chances of finding incidental but interesting results.

Combining clinical and spatial data with the unstructured clinical history allows researchers to reconstruct a full picture of each subject. This is valuable for making accurate interpretations of results, for understanding deeper and more accurately the dataset and its outliers. Participants in BRAVIZ are always linked to all their associated data, therefore they are always analyzed with respect to their whole portrait. Subjects with specific conditions can be analyzed in isolation, or attempts can be made to generalize the properties of a peculiar subject. Back and forth analysis of the data are supported by BRAVIZ with iterations to isolate a case or to generalize to the group. In fact, samples of participants or data in BRAVIZ are recognized as an essential component of the analysis. They can be created in several ways, saved into the database for future use or used instantaneously in statistical models or group data visualizations. Researchers can thus iterate through several samples, several models, several measurements, and several views, thus gaining understanding of the data-set and raising questions and hypotheses for future projects.

A log of each analysis session is automatically kept and made available through a web interface with all actions performed with the different applications used. Researchers can enhance this log by labeling the most important steps they made during a session and providing annotations. The status of an application can be reloaded at any point with a simple click of a button so that researchers are able to revisit visualization and explore different analysis paths, thus making possible to build on top of what was found with previous sessions.

In contrast to current tools, BRAVIZ enables the visualization of data from different participants and the between-participant comparisons with most associated technical issues hidden to the user. The traditional separation of workspace, with one window for medical image viewer, and a second for a spreadsheet of clinical data, the researchers manually (and repeatedly) search for correlations between both kinds of data. BRAVIZ fixed this issue of storage of individual files to manage in different softwares (access, format, and transform data are automated and hidden actions) and harnesses and proceeds data in the same workspace. It follows that users focus on the task at hand, and not on technical details.

## 7. Conclusions and future work

BRAVIZ is a tool that facilitates interactive data visualization and exploratory analysis of datasets that combine clinical and neuro-image data. It is implemented as a set of applications that can be used at different points of exploratory analysis, possibly by different members of an interdisciplinary research team. Some applications are designed to extract scalar measurements from image data, others provide detailed views of particular subjects or data types and others perform group analyses. Custom samples of subjects can be defined and used across all applications to focus on the analysis. When several applications are running together, all of them can keep focus on the same subject (Figure 9) which supports and guide the users in their understanding of how the multi-dimensional views of the same individual's data interact together.

The architecture also streamlines implementation of additional applications by encapsulating all low level data manipulation in a library. For example, BRAVIZ could easily be extended to support group based analysis like second level fMRI or VBM (Voxel Based Morphometry). In addition, several researchers have expressed interest in integrating other kinds of data, for example EEG signals or connectome graphs derived from DWI or fMRI. BRAVIZ is distributed under a LGPL license and the source code is available through the project web page http://diego0020.github.io/braviz. Additional testing with various datasets and by researchers of multiple backgrounds is required. Any assistance will be provided to anyone interested in setting up the environment and using it.

Braviz has been tested on a larger project from the Kangaroo Foundation which includes about 450 participants, complex event related fMRI paradigms, larger numbers of clinical variables, and more anatomical imaging modalities. A common concern is whether this approach will work with larger datasets, with thousands or even millions of participants. Data visualization can surprise us, but generally does not scale well; while data modeling typically does scale but cannot surprise us (Wickham and Grolemund, 2016). Therefore, one must iterate between visualization and modeling. One approach is to start exploring with a smaller subsample, and when an interesting trend is found, to try to see if generalization works for the full sample. Likewise, visualizations on a smaller subsample can be used to further understand and explore results obtained elsewhere. To this end, BRAVIZ provides the subsample mechanism.

Currently there is a trend toward moving data storage and processing to dedicated servers and providing access to users via web browsers. This allows researchers to work from any place, or even start a session in one place and finish somewhere else. Logs, variables, samples, and other analysis artifacts could be kept in a centralized location to ease data downloading from a single place. In addition, if multiple experts used the same back-end, sharing would become trivial. The back-end could be implemented via a set of dedicated servers, or it could run in a scalable cloud. Powerful data processing algorithms could also be provided to leverage high performance computing infrastructure. BRAVIZ ought to be accessible through a web interface in the future, given the potential generalization of its applicability.

The evergrowing interest in collecting data openly and making it available to the general community makes visual exploratory analysis tools like BRAVIZ become increasingly important. BRAVIZ has the ability to rapidly iterate and improve thus its future versions could contribute to increase the scientific productivity, optimally use the collected data, integrate the analysis between different variables and different users, and eventually speed-up knowledge transfer. The case studies suggested that BRAVIZ could represent a clinically relevant tool to understand brain function but also in the future to assess and help in decision making for care and therapy over lifespan.

## Author contributions

DA designed and implemented the BRAVIZ software, under the supervision of JH and JO. All three drafted the technical portions of the manuscript. CS and NC contribute with final user point of view (pediatrician and neurophysiological) along the design and prototyping process, they conduct and wrote cases studies. All authors revised and contributed to the final version of this manuscript.

## Funding

This work was developed as part of a PhD funded by the Departamento Administrativo de Ciencia, Tecnología e Innovación, Colombia (Colciencias), Grant number 528, national PhDs.

### Conflict of interest statement

The authors declare that the research was conducted in the absence of any commercial or financial relationships that could be construed as a potential conflict of interest.
